# First new species of the Neotropical genus *Lactistomyia* Melander, 1902 (Diptera, Hybotidae) from the Palearctic Region

**DOI:** 10.3897/zookeys.1272.183594

**Published:** 2026-03-10

**Authors:** Seunghun Jung, Sangil Kim, Seunggwan Shin

**Affiliations:** 1 School of Biological Sciences and Institute of Biodiversity, Seoul National University, Seoul 08826, Republic of Korea Museum of Comparative Zoology and Department of Organismic and Evolutionary Biology, Harvard University Cambridge United States of America; 2 Research Institute of Basic Sciences, Seoul National University, Seoul 08826, Republic of Korea School of Biological Sciences and Institute of Biodiversity, Seoul National University Seoul Republic of Korea; 3 Museum of Comparative Zoology and Department of Organismic and Evolutionary Biology, Harvard University, Cambridge, MA 02138, USA Research Institute of Basic Sciences, Seoul National University Seoul Republic of Korea

**Keywords:** COI barcode, Korea, new species, phylogeny, trans-Pacific distribution

## Abstract

*Lactistomyia koreana***sp. nov**., the first species of the genus *Lactistomyia* Melander, 1902, recorded from the Palearctic Region, is described based on four female and one male specimens collected in Korea. Our discovery of the *Lactistomyia* species from Korea documents an unusual trans-Pacific distribution of the genus previously known only from the Neotropical Region. The systematic affinities of the new species with the Neotropical *Lactistomyia* is supported by a phylogenetic analysis based on the mitochondrial cytochrome *c* oxidase subunit I (COI) gene sequences. An updated checklist and identification key to the world species of *Lactistomyia* is provided, and morphological characters distinguishing the new species from its congers are discussed. *Lactistomyia koreana***sp. nov**. is morphologically most similar to *Lactistomyia paranaensis* Ale-Rocha, 2008, from Paraná, Brazil, for which holotype images are presented for comparison. In addition, the generic placement of *Lactistomyia polita* Melander, 1928, from the Philippines—which was previously transferred to *Syndyas* Loew, 1857, and was given the new name, *Syndyas melanderi* Ale-Rocha, 2008—is discussed based on examination of holotype images. These findings confirm *L. koreana***sp. nov**. as the first unequivocal record of *Lactistomyia* from the Old World.

## Introduction

Hybotidae is a well-recognized family within the superfamily Empidoidea, comprising more than 1,600 extant described species worldwide (Evenhuis and Pape 2023). Species of this family are typically small, dark-coloured flies, often with raptorial hind legs, and commonly found in forest habitats with moist soil. Adults generally prey on small invertebrates, although a few species are known to visit flowers for nectarivory or palynivory (Chvála 1983; Ale-Rocha and De Freitas-Silva 2016).

The genus *Lactistomyia* Melander, 1902 (Hybotidae, Hybotinae) was established by Melander (1902), with *Lactistomyia insolita* Melander, 1902, from Brazil designated as the type species, and it is currently represented by 11 described species, all restricted to the Neotropical Region. Members of this genus exhibit typical characteristics of the family Hybotidae—such as a horizontally oriented and heavily sclerotized proboscis without pseudotracheae—that are shared with all Hybotinae except species of the genus *Euhybus* Coquillett, 1895 and members of the tribe Bicellariini Sinclair & Cumming, 2006. In addition, adults of *Lactistomyia* share diagnostic characters with other genera in the tribe Hybotini, such as holoptic compound eyes in both sexes, a stout and strongly sclerotized proboscis without pseudotracheae, and an incrassate hind femur with rows of ventral spines. The biology of *Lactistomyia* remains unknown, largely due to the scarcity of specimens and observational data (Ale-Rocha 2008).

On the Korean Peninsula, only two species of Hybotidae have been recorded to date: *Bicellaria koreana* Barták, Plant & Kubík, 2013, described from Baekdusan Mountain in North Korea (Barták et al. 2013), and *Leptodromiella crassiseta* Tuomikoski, 1932, of the subfamily Ocydromiinae, recently recorded from South Korea (Kim and Suh 2022). This limited record suggests the potential for yet undiscovered hybotid diversity in Korea, particularly when compared to adjacent countries in Northeast Asia. In this study, we describe a new species of *Lactistomyia* from Korea, representing the first discovery of the genus outside the Neotropical Region. Based on morphological examination and molecular phylogenetic analysis, we confirm the systematic placement of the new species and provide an updated key to all species of the genus *Lactistomyia*.

## Materials and methods

### Taxon sampling and morphological examination

This study is based on five specimens of *Lactistomyia koreana* sp. nov. currently deposited in the Entomology Collection at the School of Biological Sciences, Seoul National University (**SNUE**) and the Canadian National Collection of Insects, Arachnids, and Nematodes, Canada (**CNC**). The materials were collected with Malaise traps. All specimens were wet-preserved in 99% ethanol but were placed in 70% ethanol during morphological examination and subsequently returned to 99% ethanol for long-term storage. For genitalia preparations, the genitalia and adjacent abdominal segments were carefully removed using mini-dissecting scissors and macerated in a 10% potassium hydroxide solution overnight at 56 °C to dissolve soft tissues, rinsed in an 8% acetic acid solution and distilled water, and subsequently fixed in glycerin for examination and long-term storage.

Photographs of adult habitus were taken with a Canon EOS 90D camera mounted on a Stackshot Macro Rail (Cognisys Inc., USA) with an MP-E 65 mm macro lens. Terminalia images were taken with a Dhyana 400DC camera mounted on a Leica MZ12 stereomicroscope. Multiple images at different focal distances were stacked into a single composite image using Helicon Focus software (HeliconSoft, Ltd, Ukraine). Body length was measured from the base of the antennae to the apical tip of the genitalia, and wing length was measured from the radial distal tip to the base of the tegula. A transparent ruler was used for measurements under the stereoscope, except for wing veins, where the scale measurement tool in MOSAIC v. 2.3 software was employed.

Terminology for adult structures primarily follows Sinclair and Cumming (2006) and Cumming and Wood (2017), with terms for terminalia based on Sinclair et al. (2013).

### DNA extraction and phylogenetic analysis

DNA extraction was performed by grinding three legs from each specimen (except whole body of holotype and the male paratype was soaked for 3 h to preserve morphological characters) using OmniPrep Genomic DNA Purification kit (G-Biosciences, USA), following the manufacturer’s protocol with modifications. For PCR amplification, the LCO1490-HCO2198 primer pair (Folmer et al. 1994) was used to target the 658-base region of mitochondrial gene coding mitochondrial cytochrome *c* oxidase subunit I (COI). DNA amplification was performed with mixture of 2 ng of template DNA, primers and dH_2_O up to a total volume of 20 μL, added in AccuPower® PCR premix (Bioneer Inc., Korea). Thermal cycle conditions were 3 min of denaturation at 95 °C followed by 36 cycles of 95 °C for 1 min, annealing temperature at 48 °C for 1 min, 72 °C for 1.5 min then ended with 3 min of final extension at 72 °C (Lim et al. 2010). PCR success was confirmed via gel electrophoresis using 2 μL of PCR products loaded in 1.5% agarose gel. The resulting amplicons were sequenced using the ABI PRISM BigDye Terminator v. 3.1 Cycle Sequencing Kit on ABI PRISM 3730xl automated sequencers (Life Technologies, USA) at Bionics Co., Ltd (Korea). Raw sequence data were assembled into contigs and examined in Geneious Prime v. 2024.0.5. The sequences were aligned using MAFFT v. 7.490, implemented in same software with default alignment parameters (scoring matrix: 200PAM/k=2; gap opening penalty: 1.53; and offset value: 0.123) (Katoh et al. 2002; Katoh and Standley 2013). The Barcode of Life Data (BOLD, http://v4.boldsystems.org/) index numbers for each taxon included in this study are provided in the resulting phylogeny in respective tip labels (Fig. 4). Phylogenetic tree reconstruction was performed using both maximum likelihood (ML) and Bayesian inference (BI) under the GTR + G4 model as the best-fitting substitution model suggested by Modelfinder (Kalyaanamoorthy et al. 2017). We used IQ-TREE multicore v. 2.2.2.6 (Minh et al. 2020) to conduct the ML analysis, and branch support values were assessed via nonparametric bootstrap method with 1,000 bootstrap replicates. The BI analysis was carried out in MrBayes v. 3.2.7a (Ronquist et al. 2012), running four chains for 10 million generations with trees sampled every 20,000 generations. The first 2.5 million generations of each run were discarded as burn-in, and convergence of the runs was diagnosed in Tracer v. 1.7.2 (Rambaut et al. 2018).

## Results

### Taxonomy

#### Lactistomyia

Taxon classificationAnimaliaDipteraHybotidae

Melander, 1902

403DFE17-F4A6-59ED-8D4F-5D8AACD7C04B

Lactistomyia Melander, 1902: 250. Type species: Lactistomyia insolita Melander, 1902 (by monotypy).

##### Diagnosis.

**Figure 1.**
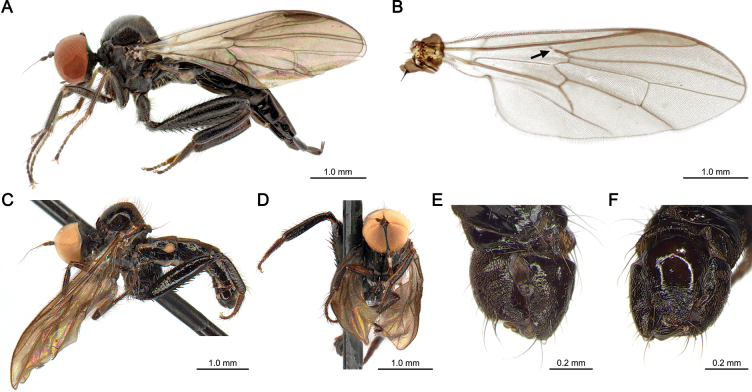
*Lactistomyia koreana* sp. nov. from Korea. **A**. Lateral habitus of the female holotype (SNUE01008); **B**. Wing venation of a female paratype (SNUE00117), with vein Rs bent at an obtuse angle and weak in its proximal half—diagnostic characters of the genus—highlighted by an arrow; **C–F**. Male paratype (CNC2153825); **C**. Lateral habitus; **D**. Frontal habitus; **E**. Male terminalia in dorsal view; **F**. Male terminalia in ventral view. Photos of male paratype habitus courtesy of Bradley Sinclair (Canadian National Collection of Insects, Arachnids, and Nematodes, Canada).

Robust, relatively large species (body at least 5 mm), thorax hunchbacked with brownish microtrichia and hair-like setae; face long, narrow; eyes holoptic in both sexes, nearly meeting or contiguous on face (Fig. 1D); antennal postpedicel ovate laterally; stylus arista-like, bare; palpus slender, flattened anteriorly, length subequal to proboscis, microtrichose with one thin seta at near apex dorsally; palpifer slender, ovate laterally and tapering anteriorly; proboscis long, projecting anteriorly with labrum sclerotized and without pseudotrachea; hind femur incrassate, with ventral spines sitting on tubercules; hind tibia tubular, curved inward; hind tarsomere 1 cylindrical, with length longer than other hind tarsomeres combined; wing vein Rs very short, bent in obtuse angle; wing veins R4+5 and M1 convergent or almost parallel near wing margin; female ovipositor withdrawn.

##### Species included.

*Lactistomyia dimidiata* (Bellardi, 1861); *L. hyalina* Bezzi, 1909; *L. insolita* Melander, 1902; *L. koreana* sp. nov.; *L. lepida* Ale-Rocha, 2008; *L. mammifera* Curran, 1931; *L. minuta* Ale-Rocha, 2008; *L. nigripes* Curran, 1931; *L. paranaensis* Ale-Rocha, 2008; *L. pulchra* Ale-Rocha, 2008; *L. serrata* Bezzi, 1909 and *L. tuberculata* Ale-Rocha, 2008.

###### Key to the species of *Lactistomyia* Melander

(modified from Ale-Rocha 2008)

**Table:** 

1	Hind legs with hind tibia tubular or slightly curved inward, without longitudinal furrow; hind femur with anteroventral and posteroventral rows of uniform spines, ventral surface wide and flattened; pterostigma prominent	**2**
–	Hind legs with hind tibia flattened anteriorly, with longitudinal furrow; pterostigma and hind femur variable	**4**
2(1)	Fore legs wholly blackish brown; hind tibia black, with brownish tint basally	***Lactistomyia koreana* sp. nov**.
–	Fore legs with femur and tibia pale brown or yellow; hind tibia extensively yellow	**3**
3(2)	Hind tibia slightly curved; hypandrium broad	***L. lepida* Ale-Rocha**
–	Hind tibia tubular; hypandrium narrow	***L. paranaensis* Ale-Rocha**
4(1)	Pterostigma infuscated	**7**
–	Pterostigma hyaline	**5**
5(4)	Prescutum without tomentum dorsally; hind femur with anteroventral and posteroventral rows of uniform spines, ventral surface wide and flattened	***L. minuta* Ale-Rocha**
–	Prescutum tomentose; hind femur with anteroventral and posteroventral rows of irregular spines, ventral surface narrow and not flattened	**6**
6(5)	Hind femur blackish brown, paler apically; wings near alula infuscate	***L. hyalina* Bezzi**
–	Hind femur wholly brown; wings tinged brown on basal half	***L. nigripes* Curran**
7(4)	Hind tibia with ventral tubercle at base	**8**
–	Hind tibia without ventral tubercle	**10**
8(7)	Wings uniformly brownish; hind femur brown with apex paler; hind tibia with sub-basal ventral tubercle minute; hypandrium with ventral lobe projecting posteriorly and apex blunt	**9**
–	Wings extensively dark brown, somewhat paler along lower margin and apically; femur wholly brown; hind tibia with greatly developed ventral tubercle; hypandrium with ventral lobe bifurcated apically	***L. mammifera* Curran**
9 (8)	Scutellar setae strong; hypandrium with ventral lobe projecting posteriorly, clavate, long and reaching base of right surstylus	***L. insolita* Melander**
–	Scutellar setae weak, short; hypandrium with ventral lobe projecting posteriorly, narrow, short with small lateral process near apex	***L. tuberculata* Ale-Rocha**
10 (7)	Thorax black with pleura brown; hypandrium with ventral process trifurcated posteriorly; right surstylus rounded	***L. pulchra* Ale-Rocha**
–	Thorax wholly brown to black; hypandrium with ventral lobe projecting posteriorly, incrassated with apex blunt; right surstylus with sclerotized dorsal cleft	***L. serrata* Bezzi**

#### Lactistomyia koreana

Taxon classificationAnimaliaDipteraHybotidae

Jung, Kim & Shin sp. nov.

FA016A59-A21A-5C0D-A5B9-D9C9B3DEE4D0

https://zoobank.org/C9A78288-6978-4867-8845-C7F1689F254B

Fig. 1

##### Type material.

***Holotype***. Korea • 1♀; Gangwon-do (prov.) Yongdae Nat. Rec. Forest, Yongdae-ri, Buk-myeon, Inje-gun; 38°14'44.5"N, 128°19'59.2"E; 13 Jun.–20 Jul. 2022; S. Jung, S. Kim, S. Shin leg.; Malaise trap; SNUE01008 (SNUE); ***Paratypes***. Korea • 2♀♀; same locality as holotype; 7 Jun.–13 Jul. 2021; S. Jung, S. Kim, S. Shin leg.; Malaise trap; SNUE00116, SNUE00117 (SNUE); Korea • 1♀; same locality as holotype; 16 May–22 Jun. 2023; S. Jung, S. Kim, S. Shin leg.; Malaise trap; SNUE02296 (SNUE); S. Korea • 1♂; Gangwon-do: Chuncheon-si, Nam-myeon, Baslan [sic], Hongcheon R., sand bar, 37°43'47"N, 127°34'35"E; 300 m; 7–24.vi.2006; P. Tripotin leg.; MT; CNC2153825 (CNC).

##### Differential diagnosis.

**Figure 2.**
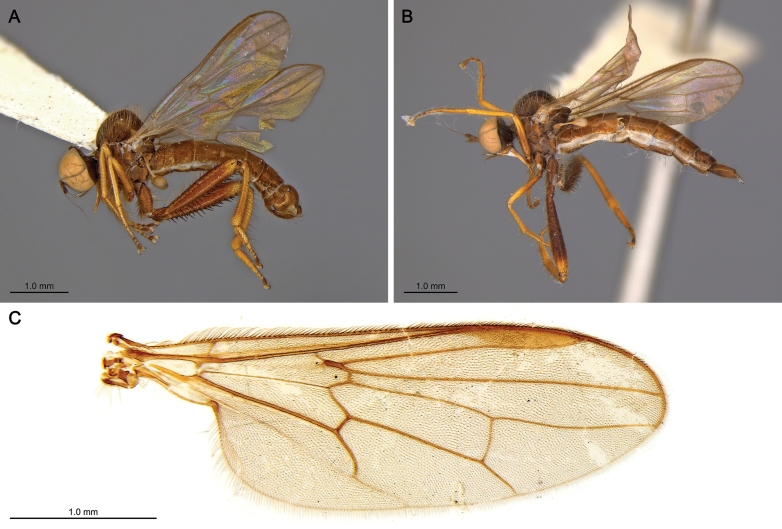
Type specimens of *Lactistomyia paranaensis* Ale-Rocha, 2008, from Brazil. **A**. Lateral habitus of male holotype; **B**. Lateral habitus of female paratype; **C**. Wing vein of a paratype. Photos courtesy of Rosaly Ale-Rocha (Instituto Nacional de Pesquisas da Amazônia, Brazil).

This species resembles *Lactistomyia lepida* Ale-Rocha, 2008, and *Lactistomyia paranaensis* Ale-Rocha, 2008 (Fig. 2), in having hind legs with a tubular hind tibia lacking an anterior longitudinal furrow. However, females of *Lactistomyia koreana* sp. nov. can be distinguished from the other two species by the combination of following characters: legs mostly blackish brown; hind legs with femur and tibia entirely black, with bluish iridescence, without pale brown ring in middle; hind femur with additional ventral spines; hind tibia slightly inflated distally.

##### Description.

**Female (Fig. 1A, B). *Head*** slightly lustrous, black with greyish microtrichia. Eyes holoptic, subdivided into dorsal section with slightly enlarged globular ommatidia and ventral section; ocellar tubercle prominent, with pair of ocellar setae thin, elongated, somewhat proclinate; postocular setae thin, black, proclinate; lower occiput with sparse, short, pale hairs. Antenna black, microtrichose except stylus; scape short, cylindrical; pedicel globular, slightly enlarged, with 2–3 thin, black, dorsal setae projected anteriorly; postpedicel ovate laterally, about twice as long as wide, yellowish basally; stylus arista-like, about five times as long as postpedicel. Face black, narrow, microtrichose; palpus slender, distal apex flattened, slightly shorter than labrum, microtrichose, with distinct black seta at tip dorsally and projected ventrally; palpifer sclerotized, bare, ovate laterally, tapered anteriorly, length subequal to palpus; proboscis blackish brown, prementum black, projected anteriorly; labrum with triangular epipharyngeal blade bifurcated at tip; hypopharynx with row of sparse spines ventrally. ***Thorax*** strongly arched, slightly lustrous, black, with brownish setulae except on dorsal portion of prescutum. Proepisternum slightly projected anteroventrally with thin setulae at anterior margin; postpronotal lobe prominent, anteroapically narrow, apex blunt. Scutum with brownish setulae, except prescutum bare, and two pairs of long, proclinate, black acrostichal seta in front of prescutellar depression; notopleuron laterally flattened, about as wide as mesopleuron in lateral view, with two strong black setae and row of sparse supra-alar setae along dorsal margin; intra-alar with a row of pale setae. Scutellum broad, with blunt apex, bearing 10 strong apical scutellar setae getting progressively shorter laterally; post-alar with one black seta. ***Legs*** slightly lustrous, black except mid legs with tarsomeres 1–2 yellow in colour; coxae microtrichose with brownish setulae and dense, black hair-like setae distally. Fore leg blackish brown; trochanter and femur with fine, short, black setae; tibia with protibial gland basally and two black setae at distal margin dorsally; fore tarsus blackish yellow, with black setae dorsally; tarsomere 1 with three distinct black setae dorsally; tarsomere 2 with distinct black seta at distal margin dorsally. Mid leg slender, blackish brown, with black setae; mid coxa with pale hair-like setae; mid tibia with two rows of four strong, black setae dorsally and ventrally, two brownish, bristle-like setae at distal margin, and one distinct long, bristle-like seta ventrally toward tarsus; mid tarsomere 1 brownish, with row of three black, bristle-like setae dorsally, and one distinct long, brown seta laterally at one-fifth from base. Hind leg with femur and tibia bluish iridescent; hind coxa with hair-like, pale setae posteriorly; hind trochanter with dense brownish hair-like setae and one short, thin, black spine ventrally; hind femur incrassated distally, with rows of long, black setae dorsally, and 20–23 black spines ventrally with two rows of five at basal half, one row of 10 distally; hind tibia slightly inflated distally, length about twice as long as tarsomeres 2–4 combined, with row of black setae dorsally and one distinct bristle-like, brown seta dorsally at distal margin; hind tarsus blackish brown, with setae black, except tarsomeres 2–3 brownish in colour; hind tarsomere 1 with brown spines ventrally. ***Wing*** (Fig. 1B) nearly hyaline with membrane tinged brown, pterostigma at end of costa wide. Alula and squama with pale hairs at margin. Cell dm length subequal to last section of wing vein M1; wing vein Rs weaker at proximal half and bent at obtuse angle; wing vein R4+5 and M1 slightly convergent near radial margin. Halter subtranslucent, blackish yellow with brownish microtrichia. ***Abdomen*** strongly sclerotized, lustrous black, bluish iridescent laterally, tubular, tapered posteriorly; tergites almost bare except tergite 1 with dense, long, pale setae projected posteriorly. Terminalia withdrawn with setae black; tergite 8 almost encircling abdomen; tergite 10 microtrichose; cercus length subequal to tergite 10 with greyish microtrichia. ***Length*** of body 5.2 mm, of wing 4.1 mm.

**Male (Fig. 1C–F)**. Similar to female, except the following: acrostichal setae on prescutellar depression and apical scutellar setae more prominent; mid tarsomeres 1–2 blackish brown. ***Abdomen*** truncated posteriorly, with brownish microtrichia dorsally; tergites 5–8 with distinct brownish lateral setae on posterior margin. Terminalia (Fig. 1E, F) asymmetric, rotated nearly 90° to the right, with strong, pale bristles on posterior margin; hypandrium broad, long, reaching the margin of the left epandrial lamella, with brownish setulae near distal margin; hypandrial lobe triangular, tapered posteriorly, short, about one-quarter length of basal portion of hypandrium, with one distinct bristle about one-third length of hypandrium; epandrial lamellae with strong preapical bristles, length about as long as hypandrial lobe; right surstylus bifurcated, with ventral lobe broad, long, and dorsal lobe short, digitiform with narrow apex. ***Length*** of body 4.2 mm, wing 3.6 mm.

##### Remarks.

Ale-Rocha (2008) proposed a species group that consists of the three Neotropical species, *Lactistomyia lepida*, *L. paranaensis*, and *L. dimidiata*, that are characterized by having hind legs lacking a conspicuously curved tibia and an anterior longitudinal furrow. Remarkably, *Lactistomyia koreana* sp. nov. exhibits these diagnostic characters and therefore can be included in this species group.

The biology of *Lactistomyia* is entirely unknown. However, based on the presence of raptorial hind legs, an anteriorly projecting labrum, and a serrated epipharyngeal blade in female specimens of *Lactistomyia koreana* sp. nov., adult flies of this genus are inferred to be predatory. These flies presumably capture prey in mid-flight, as observed in other species of Hybotinae, rather than hunting on the ground, as is typical of species in other hybotid subfamilies, such as *Tachydromia* spp., which possess a short, ventrally arched labrum (Sinclair and Cumming 2006).

##### Etymology.

The specific epithet is derived from the country of origin of the new species.

##### Distribution.

Palearctic: Korea (Gangwon-do)

### Phylogenetic analysis

In addition to the four newly sequenced specimens of *Lactistomyia koreana* sp. nov., 83 COI barcode sequences were obtained from the Barcode of Life Data Systems (BOLD) database and included in the phylogenetic analysis. These sequences represent four additional genera of the tribe Hybotini―*Euhybus*; *Hybos* Meigen, 1803; *Syndyas* Loew, 1858; *Syneches* Walker, 1852―along with one unidentified *Lactistomyia* species from Bolivia (BOLD: SICOC412-18). For outgroup selection, *Tachydromia annulimana* Meigen, 1822 (Hybotidae, Tachydromiinae) was used to root the tree, following Wahlberg and Johanson (2018), who recovered Tachydromiinae as the sister group to Hybotinae. Our final dataset comprised 87 specimens with a sequence length of 658 bp. The resulting phylogenetic analysis recovered *Lactistomyia koreana* sp. nov. [Bayesian posterior probability (BPP) = 1.00; maximum likelihood bootstrap (MLB) = 99%] and the genus *Lactistomyia* as monophyletic groups (BPP = 0.99, MLB = 51%). Other representative species of genera of Hybotinae were also largely recovered as monophyletic with strong support. However, *Lactistomyia* was nested within the genus *Syndyas*, although this relationship was weakly supported in the ML analysis (BPP = 0.76, MLB < 50%) (Fig. 4). This close relationship between the two genera is consistent with a previous morphology-based phylogenetic analysis by Ale-Rocha (2008).

## Discussion

Coquillett (1903) treated *Lactistomyia* as a synonym of *Hybos* and suggested that diagnostic characters described by Melander (1902), such as the thickened and tuberculate hind femur, were not unique to *Lactistomyia* but can also be found in other genera of Hybotinae (e.g. *Euhybus*). However, this synonymy was proposed without direct examination of *Lactistomyia* specimens. Melander (1928) subsequently resurrected the genus and described *Lactistomyia polita* from Luzon Island, the Philippines, which he considered the first Old World representative of the genus.

In the most recent revision of *Lactistomyia*, Ale-Rocha (2008) provided a redescription of the genus and clarified diagnostic characters distinguishing *Lactistomyia* from other closely related genera. These characters include a prominent basal section of wing vein M (evanescent or weak in *Syndyas*, although this character may vary among genera); base of vein Rs bent at an obtuse angle (bent at nearly a right angle in *Syndyas*); wing cell dm long or subequal in length to the distal section of vein M2 (shorter in *Syndyas*); hind femur tuberculate ventrally (tubercles usually inconspicuous or absent in *Hybos* and *Syneches*, but it also can be rather prominent in *Hybos*); hind tibia usually slightly to remarkably curved but not clavate (clavate in *Syndyas*); and hind tarsomere 1 longer than hind tarsomeres 2–4 combined (usually shorter in *Syndyas*).

**Figure 3.**
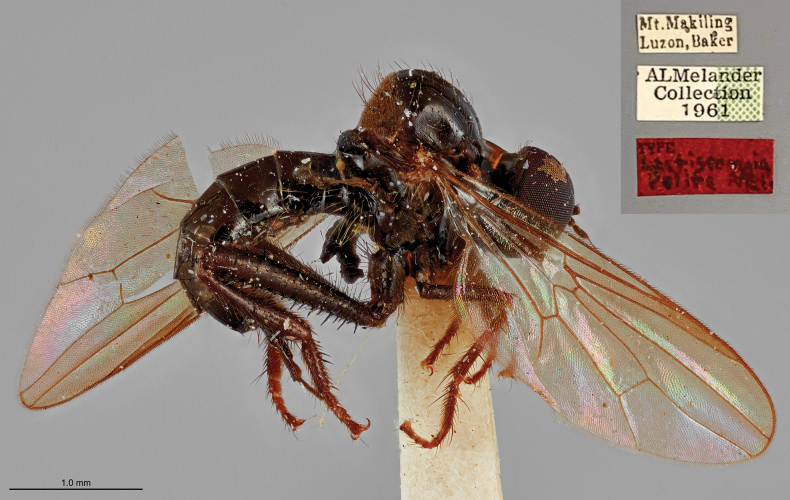
Holotype of *Lactistomyia polita* Melander, 1928, later transferred to *Syndyas* and replaced by the name *Syndyas melanderi* Ale-Rocha, 2008. Photo courtesy of Torsten Dikow (National Museum of Natural History, USA).

Based on a re-examination of the holotype, Ale-Rocha (2008) transferred *Lactistomyia polita* to the genus *Syndyas* and proposed the replacement name *Syndyas melanderi* Ale-Rocha, 2008, due to homonymy, noting that its wing and leg characters were inconsistent with *Lactistomyia*. In the present study, we also examined the holotype of *L. polita* using high-resolution photographs of the specimen housed at the National Museum of Natural History (Washington, D.C., USA) (Fig. 3), and independently confirm its placement in the genus *Syndyas* based on the following characters: Wing cell br broader than bm; basal half of bm without microtrichia; and hind legs with clavate tibia and enlarged tarsomere 1. Consequently, prior to the present study, *Lactistomyia* was known exclusively from the Neotropical Region.

**Figure 4.**
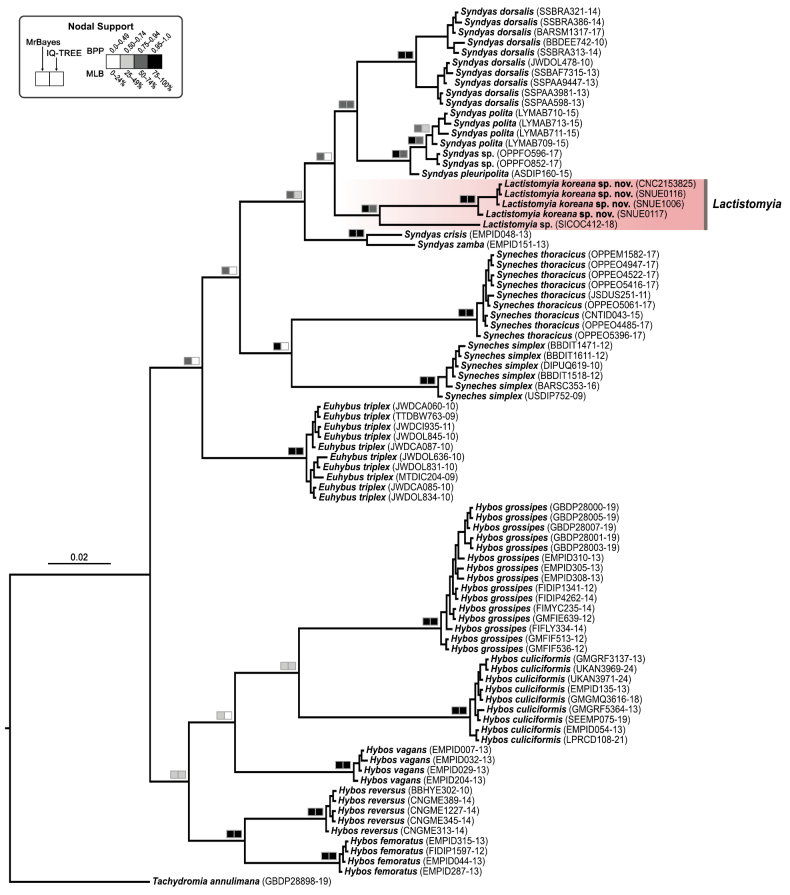
Bayesian phylogenetic tree of *Lactistomyia* and related taxa based on mitochondrial cytochrome *c* oxidase subunit I (COI) barcode sequences. Terminal labels include the taxon names with BOLD accession numbers, or specimen voucher numbers for *L. koreana* sp. nov. The clade representing *Lactistomyia* is highlighted by a red-shaded box, illustrating the sister relationship of *L. koreana* sp. nov. and *Lactistomyia* sp. from Bolivia.

In their comprehensive revision of the superfamily Empidoidea, Sinclair and Cumming (2006) recognized 14 genera within the tribe Hybotini, five of which were then known only from the Neotropical Region: *Cerathybos* Bezzi, 1909; *Euhybus*; *Lactistomyia*; *Neohybos* Ale-Rocha & Carvalho, 2003 and *Smithybos* Ale-Rocha, 2000. The discovery of *Lactistomyia* in the Palearctic Region, together with subsequent records of *Euhybus* from the Oriental regions of China (Yang and Grootaert 2007; Meilin et al. 2024), Taiwan (Liu et al. 2004) and Tibet (Wang et al. 2013), highlights that the diversity and distribution of hybotid species in East Asia remain incompletely documented and warrant further taxonomic investigation.

## Supplementary Material

XML Treatment for Lactistomyia

XML Treatment for Lactistomyia koreana

## Additional information

### Use of AI

No use of AI was reported.

### Author ORCIDs

Seunghun Jung https://orcid.org/0000-0002-8065-6696

Sangil Kim https://orcid.org/0000-0003-2758-8943

Seunggwan Shin https://orcid.org/0000-0002-4258-8661

### Data availability

The newly generated COI barcode sequences for four specimens of *Lactistomyia koreana* sp. nov. have been deposited in NCBI GenBank under accession numbers PX752067–PX752069 and PX940639.
